# Maximum Shoulder Torque and Muscle Activation During Standing Arm Flexion: Reference Data for Biomechanical and Ergonomic Applications

**DOI:** 10.3390/jfmk11010020

**Published:** 2025-12-30

**Authors:** Georgios Aronis, Michael Kurz, Florian Wimmer, Harald Hackl, Thomas Angeli, Margit Gföhler

**Affiliations:** 1 Research Unit of Biomechanics and Rehabilitation Engineering, Department of Engineering Design and Product Development TU Wien, 1060 Vienna, Austria; michael.kurz@tuwien.ac.at (M.K.); e51868066@student.tuwien.ac.at (F.W.); thomas.angeli@tuwien.ac.at (T.A.); 2 Research Unit of Machine Elements and Transmissions for Aviation, Department of Engineering Design and Product Development TU Wien, 1060 Vienna, Austria; harald.hackl@tuwien.ac.at

**Keywords:** shoulder biomechanics, joint torque, muscle activation, sagittal plane, surface electromyography, standing posture, ergonomics

## Abstract

**Objectives**: Shoulder joint strength and muscle activation during overhead reaching are critical for ergonomic task design, rehabilitation, and exoskeleton support. The objective of this study was to characterize maximum shoulder torque and flexor muscle activation profiles across functional elevation angles in healthy adult males. **Methods**: A total of 14 healthy male participants performed maximum voluntary isometric contractions at eight arm elevation angles (90–160°, sagittal plane, and standing). Shoulder torque was measured using a calibrated force sensor and normalized to each participant’s overall maximum. Electromyography (EMG) was recorded from the anterior deltoid, medial deltoid, biceps brachii, and clavicular pectoralis major; EMG for the medial deltoid, biceps brachii, and pectoralis major was normalized to muscle-specific isometric MVCs, whereas the anterior deltoid was normalized to the peak value at 90° during the main task. All EMG signals were smoothed using a 0.5 s RMS-based moving average window. Linear regression was used to analyze the torque–angle relationship, and linear mixed-effects models were used to test EMG differences across angles. Summary statistics included mean ± SD, coefficient of variation, R^2^, *p*-values (significance threshold: *p* < 0.05), Cohen’s d, and 95% confidence intervals where appropriate. **Results**: Maximum torque declined with elevation angle (y = −0.6317x + 157.21; R^2^ = 0.99), from 77.2 Nm at 90° to 43.2 Nm at 160°, with normalized values from 99.6% to 55.3%. Medial deltoid activation increased significantly with elevation (*p* < 0.001, from 87.5 ± 19.9% at 90° to 109.4 ± 25.6% at 150°), while pectoralis major declined sharply (*p* < 0.001, from 68.9 ± 24.2% at 90° to 19.8 ± 5.6% at 160°). Anterior deltoid and biceps brachii activations were high and showed no systematic change with angle (*p* = 0.37 and 0.81, respectively), remaining within approximately 95–102% and 70–85% of their reference levels across 90–160°. Normalization reduced inter-participant variability, clarifying muscle-specific trends. **Conclusions**: This study provides preliminary biomechanical reference values for shoulder torque and muscle activation across elevation angles in healthy males under isometric standing conditions, confirming an inverse torque–angle relationship and distinct muscle activation strategies at higher positions. These findings may inform ergonomic assessment and exoskeleton design, while recognizing that generalization to dynamic tasks and other populations requires caution.

## 1. Introduction

Work-related overhead tasks, such as those in construction, manufacturing, and electrical line work, significantly increase the risk of shoulder fatigue and musculoskeletal disorders, particularly rotator cuff tendinopathy, subacromial impingement, and shoulder osteoarthritis [1,2,3]. Designing effective assistive devices and ergonomic workspaces requires precise biomechanical data, particularly at high arm elevations where shoulder muscles, especially the deltoid, operate close to their physiological limits [4]. Despite this need, most existing studies investigate shoulder function only in seated or supine postures, which differ biomechanically from the standing positions typical of industrial work [5,6,7]. Furthermore, these studies generally limit measurements to arm elevation angles of 90° or below, failing to capture the demands of true overhead tasks. Hecker et al. expanded sampling up to 120° but incorporated sparse angle intervals and excluded elevations above 120°, leaving a gap in our understanding of critical overhead postures relevant for real-world activity [8].

Whole-body posture significantly influences shoulder torque production and muscle recruitment strategies. Maximum voluntary isometric contraction for shoulder flexion is typically higher when tested in a standing position, likely because the trunk and lower limbs provide additional stabilization [9,10,11]. Nevertheless, almost all available data on isometric shoulder torque and electromyography (EMG) remain derived from tests performed in seated or supine postures and at only a subset of possible elevation angles, often at or below 120 °, and even standing studies rarely assess postures at greater elevations [8,11,12].

Importantly, to the authors’ knowledge, comprehensive mapping of shoulder torque production and associated muscle activation across a continuous range of overhead elevation angles in the standing position has not been systematically reported in the literature, with most studies assessing strength or activity at discrete or limited joint positions. Such information is critical for both understanding the upper limits of human arm elevation strength and for optimizing the design of upper-limb assistive exoskeletons and overhead workspaces, which must account for the reduced mechanical advantage and increased muscle load at high elevation angles [13]. There is evidence that shoulder muscle synergies and activation strategies change as arm elevation increases, but these biomechanical patterns remain incompletely characterized for isometric tasks in a standing posture, particularly across the full range of sagittal elevation angles required for real-world activities.

In this context, the primary mechanical outcome of interest is the decline in maximum shoulder flexion torque with increasing arm elevation, reflecting the loss of mechanical advantage in overhead postures. The EMG analysis focuses on four key muscles selected for their distinct roles in sagittal elevation: the anterior deltoid as the primary flexor, especially at mid-range angles [8,14,15]; the medial deltoid, which becomes dominant in generating torque as the arm exceeds shoulder height [8,14,16]; the clavicular pectoralis major, which contributes to flexion at lower angles but loses mechanical effectiveness near end-range elevation [14,15]; and the biceps brachii, a biarticular muscle assisting flexion and glenohumeral stability under load [15,17]. Investigating these muscles together allows for characterizing how activation shifts among prime movers and synergists across the full range of overhead elevation.

The present study addresses these gaps by systematically measuring maximum isometric shoulder torque and the electromyographic activity of selected prime movers and important synergists or stabilizers at eight distinct arm elevation angles (90–160°, every 10°) in healthy adults, all assessed while standing. This dataset offers high-resolution, angle-specific torque and EMG data in the sagittal plane under conditions mimicking overhead occupational maximum voluntary contraction. The aims were to (1) describe the torque profile and muscle activation patterns as a function of elevation angle during standing rope-pull tasks and (2) discuss the ergonomic implications for exoskeleton development and workspace design. Based on established biomechanical principles, we hypothesized that maximum shoulder torque would decrease with increasing arm elevation angle. Given limited data on moment arms, length–tension relationships, and EMG at high elevations, we expected muscle-specific differences in activation profiles across the range but treated these EMG patterns as exploratory. These insights may inform novel strategies to reduce musculoskeletal injury risk and optimize human performance in overhead work contexts.

## 2. Materials and Methods

### 2.1. Participant Characteristics

A total of 14 healthy male individuals (age: 37.1 ± 12.6 years; height: 181.0 ± 6.9 cm; weight: 87.9 ± 15.9 kg) were recruited. All participants had no history of shoulder injury. Informed consent was obtained from all participants, and the study was conducted in accordance with the Declaration of Helsinki. The study protocol was approved by the Ethics Committee of TU Wien (approval code: 089_FT112025_TUWREC, date of approval: 24 November 2025).

The sample size was determined based on a power analysis for repeated measures designs. With 8 within-subject measurements (arm elevation angles), α = 0.05, and assuming large effects (Cohen’s f = 0.40), a minimum of *n* = 13 participants was required to achieve 80% power. To enable counterbalancing of testing order (half beginning at 90° progressing to 160° and half beginning at 160° progressing to 90°), we recruited n = 14 participants (7 per order condition), which provides 86.4% power to detect large effects. This sample size is consistent with comparable shoulder biomechanics studies using within-subjects designs: Krainak et al. (*n* = 21) [18] and Beardsley et al. (*n* = 12) [19].

### 2.2. Testing Setup and Procedure

**Figure 1:**
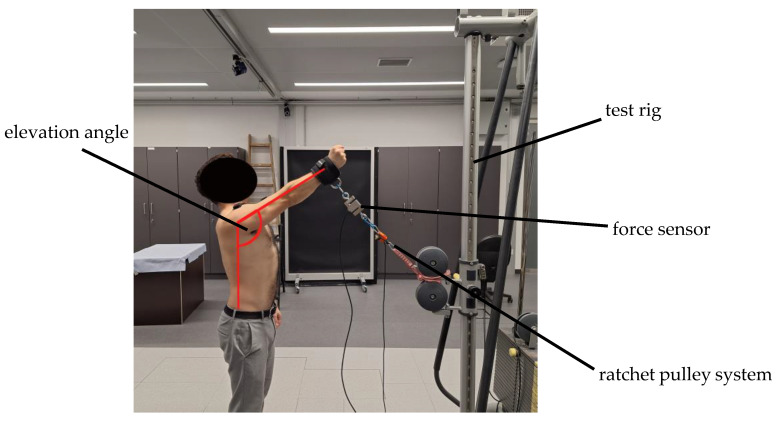
Subject performing isometric shoulder elevation, pulling against resistance.

Participants were positioned in front of a stationary test rig so that their fully extended arm was aligned perpendicular to the line of applied force (see Figure 1). The setup was individually adjusted based on each participant’s shoulder height and arm length to ensure consistent alignment. All measurements were performed with the participant’s dominant arm. The hand was positioned with the thumb pointing upward, palm facing medially, and the fist closed to standardize wrist and forearm posture.

A wrist cuff was secured to the participant and connected to one side of a tension force sensor. The opposite side of the sensor was attached to a ratchet pulley system, whose free end was mounted to the vertical frame of the test rig. The pulley attachment point on the rig could be adjusted vertically between 20 and 220 cm above the ground to accommodate different arm elevation angles and participant anthropometrics.

As the target arm elevation angle changed, both the pulley attachment height and the effective rope length were adjusted so that the participant’s arm maintained the desired elevation angle (90–160° relative to vertical) and a consistent 90° angle between the arm and the line of action of the rope. These angles were verified using a 30 cm universal goniometer (66fit Ltd., Spalding, UK; 1° resolution). After each setup adjustment, participants rested for one minute before the next trial, with setup adjustments taking approximately two minutes per angle.

At each tested angle, participants performed two maximum isometric shoulder flexion efforts by pulling against the rope, separated by a 20 s rest. Each contraction lasted five seconds, during which standardized verbal encouragement was provided. The order of testing was counterbalanced across participants, with half performing trials from 90° to 160° and the other half from 160° to 90°, to minimize potential order effects or bias. The procedure was repeated for all tested elevation angles.

### 2.3. Torque Calculation

Joint torque was calculated as the product of the measured force and the participant-specific lever arm, defined as the distance between the estimated centre of the glenohumeral joint and the rope attachment point at the wrist. The glenohumeral joint center was estimated using a regression-based method from palpable scapular bony landmarks as described by Meskers et al., consistent with recommendations for upper-limb kinematic modeling [20]. Lever arm length was then defined as the straight-line distance from this estimated joint center to the wrist cuff and measured once per participant using a tape measure (1 mm resolution), yielding a mean lever arm of 0.517 ± 0.034 m. Small errors in joint center location or lever arm length translate linearly into torque error; based on validation studies, regression-based glenohumeral joint center estimation yields position errors on the order of 5–10 mm, while tape-based linear measurements show approximately 1% error compared to 3D scanning gold standards. Combining these sources conservatively (10 mm GH center uncertainty + 5 mm tape measurement error) gives a maximum lever arm uncertainty of 15 mm. For our mean lever arm of 0.517 m, this corresponds to 0.015/0.517 ≈ 3% error in torque at the mean maximal torque of 77.1 Nm. Because this uncertainty is systematic within each participant, constant across elevation angles, and torques were normalized to each individual’s maximum, its influence on the within-subject torque–angle relationship is negligible [20,21].

Since the applied force was maintained perpendicular to the lever arm throughout testing, no angular correction was necessary. The maximum torque for each trial was calculated as follows:

$$T_{ext}=F_{max}L$$

where $F_{max}$ is the maximum mean force over the 0.5 s interval (N) and *L* is the lever arm length (0.517 ± 0.034 m).

Net shoulder torque was computed as follows:

$$T_{net}=T_{ext}+\;T_{grav}$$

Gravitational torque was estimated using Winter’s anthropometric tables [22], with the arm modelled as three segments (upper arm: 2.8% BW, CoM 43.6% of length; forearm: 1.60% BW, CoM 43.0%; hand: 0.60% BW, CoM 50.6%), scaled individually to each participant’s body mass and height. Gravitational torque at elevation angle θ was calculated as follows:

$$T_{grav(\theta )}\;=\;\Sigma_{i}\;m_{i}\;g\;d_{i}\;sin(\theta )$$

where $m_{i}$ is segment mass, $d_{i}$ is distance from glenohumeral joint to segment center of mass, g = 9.81 $m/s^{2}$, and θ is arm elevation angle from vertical.

For a representative participant (87.9 kg, 1.81 m), gravitational torque at 90° elevation was 13.5 Nm, representing 18% of mean maximal net torque (77.1 Nm). Winter-based segment parameters typically differ from imaging estimates by 5–10%; a 10% error in gravitational torque would alter net torque by <2% though this could reach ~4% at lower external torque levels at higher elevation angles.

### 2.4. Sensor Placement

Prior to data collection, participants performed a standardized warm-up. Surface EMG sensors (Delsys Trigno Avanti/Mini, Delsys Inc., Natick, MA, USA) were applied to the anterior deltoid, medial deltoid, clavicular part of pectoralis major, and biceps brachii on the dominant side. Sensor placement followed SENIAM guidelines [23]. All electrode sites were carefully prepared with isopropyl alcohol to promote low electrode–skin impedance and high-quality recordings. The Trigno Avanti/Mini sensors, which use parallel bar electrodes with an inter-electrode distance of approximately 10 mm, were oriented along the presumed muscle fiber direction and positioned according to SENIAM recommendations to minimize crosstalk from adjacent muscles

### 2.5. Data Collection and Processing

Force was measured using a Sauter CS150 3P1 (Kern & Sohn GmbH, Balingen, Germany) force sensor with a sampling frequency of 1000 Hz. Data acquisition and visualization were performed in LabVIEW (National Instruments, Austin, TX, USA), and further analysis was conducted in Microsoft Excel. A 0.5 s window was selected to quantify the steady-state maintenance phase of the isometric contraction and minimize the influence of transient signal spikes or artifacts, a method shown to yield higher reliability for MVC estimation compared to shorter windows [24,25]. The choice of two 5 s MVICs with 20 s rest between trials at each angle was based on pilot testing in our laboratory and on previous shoulder strength protocols indicating that rest periods of at least 20 s between 5 s MVICs are sufficient to reproduce maximal efforts without systematic fatigue. Across the eight elevation angles, participants were given at least one minute of seated rest between angles to further reduce cumulative fatigue [26]. Force signals were filtered with a centered moving average algorithm with a 0.5 s time window to reduce noise. Surface EMG signals were recorded at a sampling rate of 2148 Hz and processed in Python (version 3.12.9) using the EMGFlow package. Surface EMG was preprocessed using a standard pipeline consisting of bandpass filtering, rectification, and RMS smoothing. Signals were first bandpass filtered between 20 and 450 Hz and notch filtered at 50 Hz to attenuate movement artifacts and power-line noise. The filtered signals were full-wave rectified, and an RMS envelope was then computed using a 0.5 s moving window, yielding a time-dependent EMG envelope with a 0.5 s maximum for each sample point. As a sensitivity analysis, group-mean normalized EMG for the anterior deltoid, medial deltoid, pectoralis major, and biceps brachii was recomputed using RMS windows of 50, 100, and 200 ms and compared with the 500 ms window across all elevation angles. All trials were visually inspected in both force and EMG traces, and trials with clear artifacts (e.g., force drops, abrupt spikes, or EMG saturation due to motion or poor contact) were excluded from further analysis. For the remaining valid trials, the peak value from the smoothed trace was extracted and normalized to the corresponding MVC.

After maximum torque values were calculated using the peak force values at each elevation angle, these torque values were normalized to each participant’s highest torque observed across all tested angles (found at 90° in 12 out of 14 participants, at 100° for one participant, and at 110° for another). This yielded a dimensionless normalized torque, expressed as a percentage of each individual’s maximum. Surface EMG signals were normalized to muscle-specific reference contractions. For medial deltoid, biceps brachii, and pectoralis major, EMG at each elevation angle was divided by the peak EMG obtained from separate isometric MVC trials in standardized postures for each muscle. For anterior deltoid, EMG was normalized to the peak value recorded at 90° shoulder flexion during the main testing protocol, because this contraction was mechanically identical to the anterior-deltoid MVC task and consistently yielded higher EMG amplitudes than the separate anterior-deltoid MVC trials, indicating that the latter were likely submaximal. No separate test–retest session was conducted in this study; however, prior work using comparable isometric shoulder strength protocols and 5 s MVIC EMG normalization procedures has reported good to excellent within-session and between-session reliability (ICC typically ≥0.85) [27].

### 2.6. Statistical Analysis

Descriptive statistics (mean ± SD) were computed for maximum torque, normalized torque, and normalized EMG activity at each arm elevation angle. To characterize the torque–angle relationship, normalized torque values were analyzed at both the individual and group levels using linear regression. For each participant, a simple linear regression of normalized torque versus elevation angle was fitted to obtain an individual slope (percentage points of normalized torque per degree). At the group level, a linear mixed-effects model with normalized torque as the dependent variable, elevation angle as a continuous fixed effect, and random intercepts and slopes for participants was used to estimate the average torque–angle slope and its 95% confidence interval (CI) and *p*-value. For graphical illustration only, a linear regression of group-mean normalized torque versus elevation angle was also computed. The coefficient of variation (CV) was calculated for both normalized and absolute torque to assess inter- and intra-subject variability in shoulder strength. For selected within-subject contrasts (e.g., 90° vs. 160°), standardized effect sizes were calculated as Cohen’s d for paired samples together with 95% confidence intervals. For torque and normalized EMG, between-subject variability at each elevation angle was summarized using the standard deviation across participant.

To examine effects of arm elevation on muscular activation, linear mixed-effects models were fitted to normalized EMG values for each muscle, with elevation angle as a fixed effect and participant as a random effect. Models were fitted in Python using the statsmodels MixedLM implementation with restricted maximum likelihood (REML) estimation, and Wald z-statistics with two-sided *p*-values were reported for the fixed effect of angle.

To evaluate potential order or fatigue effects, the testing order (90°→160° vs. 160°→90°) was included as a between-subject factor. Mean normalized torque across all elevation angles was compared between order groups using an independent samples *t*-test (*p* = 0.03), and separate linear regressions of the normalized torque versus elevation angle were computed for each order group to examine whether testing order altered the torque–angle relationship.

## 3. Results

### 3.1. Normalized Shoulder Flexion Torque vs. Elevation Angle

**Figure 2:**
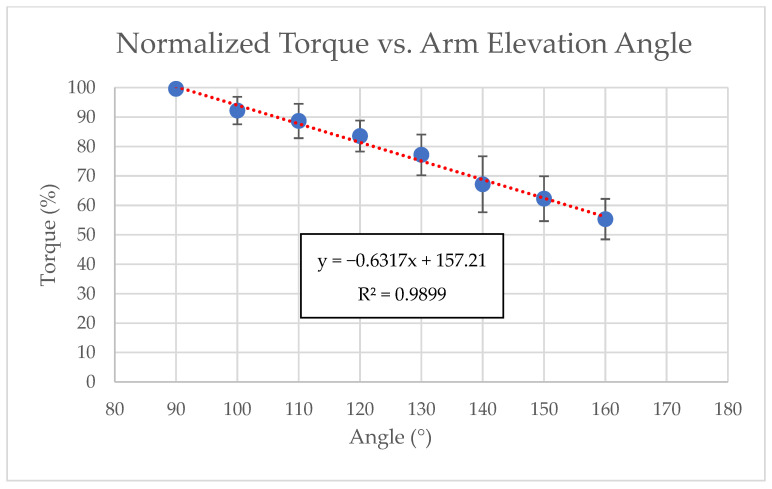
Normalized shoulder flexion torque versus arm elevation angle. Points show group-mean normalized torque at each elevation angle, and the solid line shows the linear regression trendline fitted to the means (equation and R^2^ shown in the panel).

**Figure 3:**
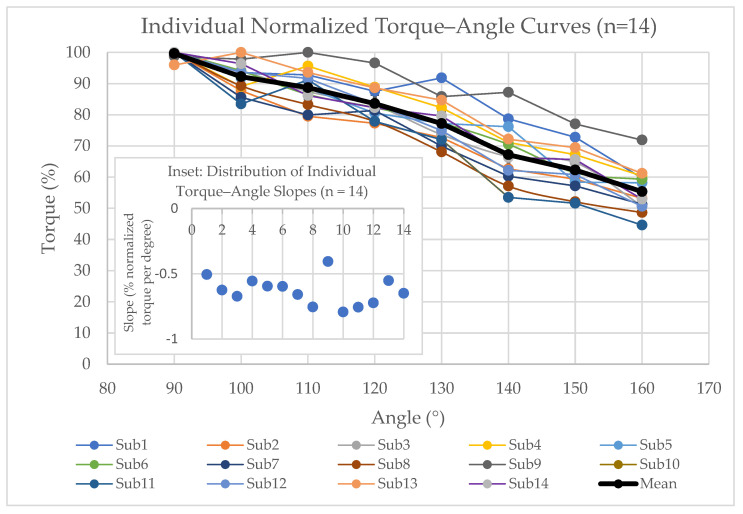
Individual normalized shoulder flexion torque–angle curves. Thin lines show individual normalized torque–angle curves for all 14 participants; the thick black line and error bars show the group mean ± 95% CI across subjects at each elevation angle. The inset displays the distribution of subject-level regression slopes for normalized torque versus elevation angle (mean −0.63% per degree, with 95% CI −0.69 to −0.57), illustrating consistently negative slopes across participants.

The normalized maximum shoulder flexion torque decreased almost linearly as arm elevation increased from 90° to 160°. In the linear mixed-effects model, normalized torque declined on average by −0.63 percentage points per degree (95% CI −0.69 to −0.57, with *p* = 1.1 × 10^−11^), confirming a robust group-level, negative torque–angle relationship. Individual subject regressions also yielded consistently negative slopes, ranging from −0.79 to −0.41 percentage points per degree (mean −0.63), indicating relatively uniform declines across participants. Figure 2 shows the group-mean normalized torque at each elevation angle together with the fitted linear regression trendline (R^2^ = 0.99). Figure 3 presents the individual normalized torque–angle curves for all 14 participants (thin lines) together with the group mean and its 95% CI at each elevation angle (thick line with error bars); the inset displays the distribution of individual regression slopes (mean −0.63% per degree, with a 95% CI −0.69 to −0.57).

The mean normalized torque at each elevation angle, calculated as the average of all participants’ peak normalized values, decreased consistently with arm elevation. At 90°, the mean normalized torque was 99.6 ± 1.1% of the maximum, indicating very low interindividual variability, which can be attributed to the fact that most subjects achieved their maximum torque at this angle. At higher angles, greater variability was observed, with the mean at 140° measuring 67.2 ± 9.5%. Full details for all elevation angles are provided in Table 1. The within-subject decrease in normalized torque from 90° to 160° was 44.3 ± 7.5%, corresponding to a very large effect size (paired Cohen’s d = −5.94, with a 95% CI −8.20 to −3.68). These findings collectively characterize the torque–angle relationship during maximum isometric shoulder flexion at high arm elevations, providing reference values for biomechanical modeling and rehabilitation applications.

Participants tested from 90°→160° exhibited slightly higher mean normalized torque across angles than those tested from 160°→90° (*p* = 0.03), indicating a modest order effect. However, separate linear fits of normalized torque versus angle for the two order groups yielded very similar slopes (−0.69 vs. −0.57, with both R^2^ > 0.98), and the individual slope distribution (Figure 3, inset) showed that all participants had negative slopes of comparable magnitude, suggesting that testing order did not meaningfully alter the torque–angle relationship.

### 3.2. Actual Shoulder Flexion Torque with Elevation Angle

The actual (absolute) maximum shoulder flexion torque decreased as arm elevation increased, paralleling the trend observed in normalized data. Mean torque values ranged from 77.24 ± 13.8 Nm at 90° to 43.19 ± 10.8 Nm at 160°. The coefficient of variation (COV) for actual torque was substantially higher than for normalized data, spanning 17.9% to 25.9% across elevation angles, which is indicative of considerable between-subject variability in absolute shoulder strength, as routinely reported in the literature. For comparison, COV for normalized torque was markedly lower (1.1% to 14.2%) at corresponding angles. The ratio of mean normalized to mean actual torque remained stable across angles (1.278 to 1.293), reflecting consistency in normalization and dataset structure. The high absolute COV values underscore the influence of anthropometric, physiological, and demographic factors on maximum isometric torque, while normalization reduces this variability and enables clearer group-level comparison.

### 3.3. Muscle Activation Profiles During Isometric Shoulder Flexion

EMG activity of the anterior deltoid, medial deltoid, biceps brachii, and clavicular part of the pectoralis major was measured while participants performed maximum voluntary isometric shoulder flexion at arm elevation angles from 90° to 160°. Table 2 presents the mean ± SD of normalized EMG activity for each muscle and elevation angle, expressed as a percentage of its muscle-specific reference contraction (MVC for medial deltoid, biceps brachii, and pectoralis major, and the 90° task-specific reference for anterior deltoid).

**Figure 4:**
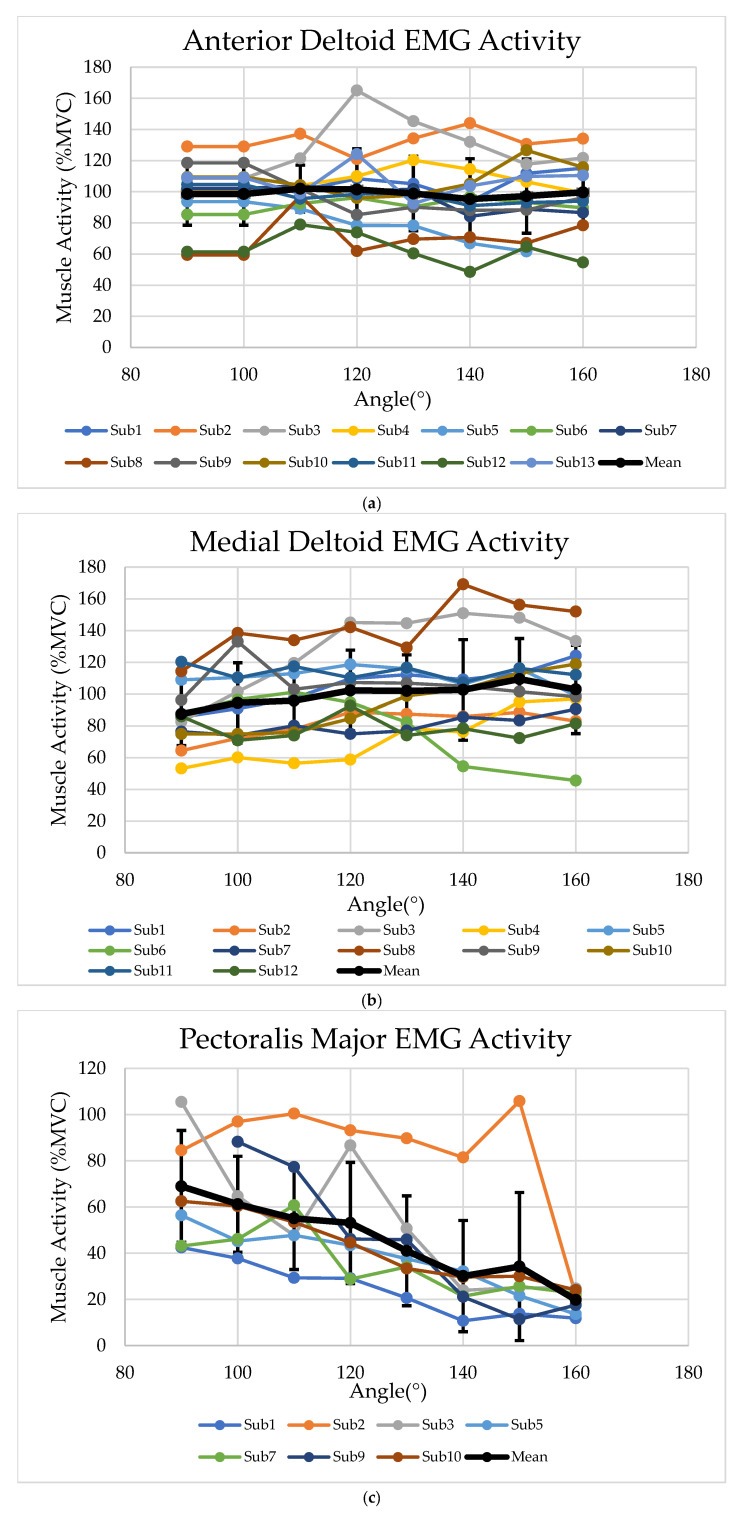

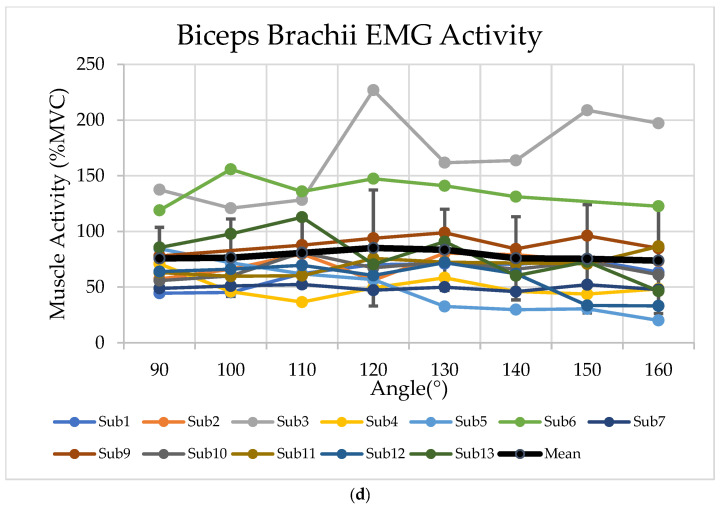
Subject-level normalized EMG across arm elevation angles (90–160°). Panels show normalized EMG for the (**a**) anterior deltoid, (**b**) medial deltoid, (**c**) biceps brachii, and (**d**) clavicular pectoralis major. Thin lines represent individual participants, and the thick line shows the group mean. EMG for medial deltoid, biceps brachii, and pectoralis major was normalized to muscle-specific MVCs, whereas anterior deltoid EMG was normalized to the peak value at 90° during the main task.

The anterior deltoid demonstrated consistently high activation at all elevation angles (approximately 98–102% of its reference level), with statistical analysis confirming that normalized EMG remained stable as the angle increased (linear mixed model, with *p* = 0.371). The biceps brachii also exhibited no significant change in EMG with elevation (*p* = 0.811), with mean values between about 74% and 86% of MVC across angles. In contrast, medial deltoid activation increased significantly with arm elevation, from 87.5% at 90° to 109.4% at 150° (*p* < 0.001). Pectoralis major showed a declining activation profile from 68.9% at 90° to 19.8% at 160° (*p* < 0.001). These trends were evident across individual participants and are depicted in Figure 4. Effect-size analyses for the 90° versus 160° contrast showed negligible changes for the anterior deltoid (Cohen’s d = −0.02, with 95% CI −0.29 to 0.25) and biceps brachii (d = −0.06, with 95% CI −0.33 to 0.21), a moderate but imprecise increase for medial deltoid (d = 0.54, with 95% CI −0.26 to 0.83), and a large decrease for pectoralis major (d = −2.08, with 95% CI −2.55 to −1.60).

Sensitivity analyses showed that shortening the RMS window (50–200 ms) produced very similar normalized EMG–angle curves for all four muscles. For anterior and medial deltoid as well as biceps brachii, mean differences relative to the 500 ms window were generally below about 5–8% at all angles, and the direction of the angle effect (stable activation for anterior deltoid and biceps and increasing activation for medial deltoid) was unchanged. For pectoralis major, shorter windows resulted in somewhat larger percentage differences, up to roughly 20% at higher elevations where absolute EMG amplitudes were low, but the progressive decrease in normalized EMG with angle was preserved.

Standard deviations for all muscles ranged from approximately 15.1% to 41.9%, reflecting moderate to pronounced inter-subject variability, most notable at higher elevation angles. Trials with clear artifacts in the force or EMG traces (e.g., premature force decline, abrupt spikes, and electrode detachment) were excluded from analysis. The number of valid participants contributing EMG data for each muscle and elevation angle is shown in Table 2. The combination of linear mixed-effects models and subject-level EMG trajectories (Figure 4) indicates that the main angle-dependent patterns were broadly consistent across participants, with no extreme outliers.

## 4. Discussion

In this study, maximum glenohumeral joint torque decreased progressively as arm elevation angle increased, with the mean value falling from 77.24 Nm at 90° to 43.19 Nm at 160°. This downward trend was evident for all participants, although standard deviations ranged from 13.81 Nm to 10.84 Nm. Normalizing the torque data to each participant’s personal maximum maintained the clear decline with increasing elevation angle. Specifically, average normalized values were 99.62% at 90° and 55.33% at 160°. This normalization also reduced variability between participants, resulting in more consistent response patterns across the group [28].

### 4.1. Interpretation of Torque–Elevation Dynamics

The analysis of the normalized torque data revealed an almost perfectly linear decline across the full range of tested elevations, which can be fit with a simple equation (y = −0.6317x + 157.21), where y is normalized torque and x is arm elevation angle. The high R^2^ value of 0.99 indicates that the nearly linear model explains nearly all of the variance in torque as a function of elevation angle. This linear pattern is broadly consistent with prior reports on shoulder flexion strength in both standing and seated positions. Bober et al. investigated shoulder flexion from −45° to 120° and found an approximately linear decrease in torque from lower to higher elevation angles [12], though absolute values differ slightly due to postural stabilization requirements and the seated testing posture used [11].

### 4.2. Muscle Activation Patterns Across Elevation Angles

Muscle activation data indicated that the anterior deltoid, the principal flexor of the shoulder in the sagittal plane, maintained consistently high and nearly constant EMG levels through the 90–160° elevation range, with mean values tightly clustered between 95% and 102% of MVC a slight peak at 110° [29]. This plateau was expected based on anatomical and EMG studies, which consistently identify the anterior deltoid as the major contributor to shoulder flexion across these angles [14,16]. The medial deltoid demonstrated a marked elevation-dependent increase, rising from around 88% at 90° to more than 109% of MVC at 150°, reflecting its expanding role in torque generation and stabilization as the arm elevates [8]. Biceps brachii EMG remained in the approximate 74–85% of MVC range without a systematic trend across angles, supporting a primarily stabilizing role rather than a major contribution to shoulder flexion torque. According to Landin et al., during shoulder flexion with the arm extended and elevation above 90°, the biceps brachii has negligible impact on shoulder flexion torque; thus, its persistent EMG activity likely reflects a stabilizing role at the glenohumeral joint rather than contributing to force production in shoulder flexion [17]. In contrast, pectoralis major revealed a pronounced decrease with increasing elevation, dropping from about 69% of MVC at 90° to nearly 20% at 160°. The decreased EMG activity of pectoralis major with increasing shoulder elevation is further supported by Ito et al., reporting a marked reduction in activation as arm elevation angle increased [30]. Standard deviations for EMG ranged from 5.6% to 52.1%, reflecting notable inter-individual variability in muscle recruitment, especially at higher angles. The high variability in muscle activation values may reflect individual neuromuscular strategies or differences in muscle morphology and sensor placement, as noted by Ashworth et al. [31].

Importantly, the EMG processing choices were evaluated with a sensitivity analysis that compared RMS windows of 50, 100, 200, and 500 ms. The small amplitude differences and unchanged angle-dependent patterns for anterior and medial deltoid, biceps brachii, and pectoralis major indicate that the relatively long 500 ms RMS window primarily reduces high-frequency variability without biasing EMG amplitudes or altering the interpretation of muscle activation across elevation angles.

### 4.3. Mechanistic Insights: Torque–Activation Relationships

The results are consistent with a logical, though not perfectly proportional, relationship between mechanical torque production and muscle activation across elevation angles. As elevation increases and mechanical disadvantage grows, maximum torque output declines, yet EMG activity remains high or even increases (except pectoralis major). This aligns with established biomechanical principles: at elevated ranges, muscles may require greater neural drive to maintain force, as their moment arms decrease and length–tension relationships become less favorable. This reduced mechanical efficiency is expected to increase energy cost and accelerate fatigue, and experimental and modelling work suggests that the nervous system may limit force production in these positions to conserve energy [32,33].

This pattern is explained through several key factors. As the arm approaches extreme elevation angles, deltoids fibres are less likely to operate at their optimal length for force production due to muscle shortening during concentric contraction [34]. Even if the moment arm remains relatively consistent within certain ranges [15,34], suboptimal fibre length at end-range reduces force output, requiring greater neural activation to compensate [33]. At high elevation angles, shoulder muscles, especially the deltoids and components of the rotator cuff, are increasingly engaged to stabilize the glenohumeral joint in addition to generating movement torque, which can elevate EMG amplitude beyond levels expected from torque requirements alone [35]. Meanwhile, activation of other flexor muscles such as pectoralis major decreases at high elevation angles due to biomechanical disadvantage and suboptimal lines of pull. This trend is consistent with biomechanical limitations and loss of flexion moment arm at higher angles. Ackland et al. further demonstrate that both the moment arm and force-generating capability of the pectoralis major are significantly diminished above shoulder height, indicating its contribution to sagittal plane flexion torque becoming progressively less as elevation increases [15]. At the same time, the role of stabilizers and extensors such as the subscapularis, infraspinatus, and teres minor may become increasingly pronounced. These muscles contribute actively and passively to resisting excessive humeral translation and help maintain joint integrity when stability demands are elevated [36].

The anterior deltoid remains highly activated as it compensates for declining input from synergists, explaining the mismatch between EMG activity and net torque production. Overall, the mismatch between declining torque and sustained EMG activity at higher angles reflects the combined influence of muscle architecture, leverage mechanics, compensatory activation for stabilization, and shifting synergist muscle roles, as supported by prior isometric studies and musculoskeletal models [15,33,34,35,36].

### 4.4. Limitations

A significant limitation is the absence of EMG recordings from all flexor and stabilizing muscles involved in shoulder elevation, especially the coracobrachialis and deeper portions of the rotator cuff. This lack of data on secondary contributors restricts comprehensive analysis of muscle coordination, particularly at high overhead positions where their stabilizing influence may be critical. Additionally, the modest sample size and the technical difficulties inherent in joint angle measurement and EMG sensor placement can influence the precision and generalizability of our findings, and the restriction to a healthy, male sample further limits extrapolation to females, older adults, and clinical populations. Standard anthropometric models and torque calculation assumptions, while widely used, may not fully account for individual anatomical variability and complex biomechanics [22], potentially introducing further uncertainty in interpreting group-level results. Furthermore, EMG amplitudes depended on the chosen normalization procedures and on the 500 ms RMS smoothing window; although sensitivity analyses with shorter windows indicated that the main angle-dependent patterns were robust, absolute activation levels and inter-subject variability should be interpreted in light of these processing choices and the isometric standing task context. Finally, no dedicated test–retest reliability protocol was performed in this study, so reliability inferences rely on published ICC values for comparable shoulder MVC protocols rather than on new estimates from our dataset.

### 4.5. Implications and Applications

The progressive decline in shoulder flexion torque with increasing elevation angle, combined with persistent or rising activation in several muscles, suggests elevated relative muscular demand at higher arm positions under isometric standing conditions. For exoskeleton and ergonomic design, these results can be used as torque ‘envelopes’ across elevation angles by considering the mean normalized strength curve together with its 95% confidence intervals at each angle, rather than relying on single point estimates. Designers should also account for the fact that these envelopes are derived from a small sample of healthy males and may underestimate variability and peak demands in other populations or dynamic work tasks. The distinction between external torque, which may decrease at higher elevations due to shorter load moment arms, and internal muscular effort, which can remain high because of biomechanical disadvantage, compensatory stabilizer recruitment, and diminished mechanical efficiency, has direct relevance for optimizing injury-prevention strategies, informing return-to-work criteria and guiding the development of targeted rehabilitation protocols. In assistive technology, such as passive exoskeletons, support profiles should be matched to the continuous decline in strength and elevated muscular demand that occur at higher arm positions, especially to reduce fatigue and improve safety during repetitive overhead tasks. Prior studies confirm that individually tailored, muscle-targeted support can reduce effort and discomfort and increase endurance across various occupational and clinical settings [37,38]. Overall, this work provides practical reference values and physiological insight applicable to a broad spectrum of fields where elevated arm postures are frequent.

### 4.6. Future Directions

While these results clarify trends in shoulder torque production and muscle activation across elevation angles, several methodological and contextual factors must be acknowledged. Future research should expand to dynamic tasks and more diverse populations to increase the relevance and generalizability of findings to real-world activities. However, even with advanced protocols, it is not feasible to record EMG from all major and minor shoulder flexors using surface electrodes because many muscles, such as the coracobrachialis or deep rotator cuff, are inaccessible non-invasively, restricting analysis of whole-muscle coordination. Thus, results from surface EMG studies should be interpreted in light of these technical constraints [39]. In addition, further mechanistic investigations are needed to clarify why muscle activation measured by EMG and produced torque sometimes diverge at high arm angles. The EMG–torque dissociation may result from shifting muscle leverage, length–tension effects, recruitment of deep or synergistic muscles, or fatigue. A deeper understanding will benefit both biomechanical theories and practical interventions. Additionally, exploring shoulder strength and muscle activation patterns in more planes of motion, not just the sagittal, would further enhance the relevance for multi-directional tasks experienced in daily life and occupational settings. Taken together, these directions should underpin future work aimed at comprehensive understanding of shoulder biomechanics and muscle function in realistic human activity contexts [40].

## 5. Conclusions

This work demonstrates an almost linear decline in maximum shoulder flexion torque with increasing arm elevation during isometric standing tasks in healthy males. In parallel, muscle activation shows distinct patterns: activation remains high and relatively stable for the anterior deltoid and biceps, rises for the medial deltoid, and declines substantially for the pectoralis major at higher elevation angles. Together, these findings provide preliminary clinical and ergonomic benchmarks for isometric overhead postures and may help inform support strategies for real-world overhead activities, including rehabilitation, injury prevention, and assistive technology development, while recognizing that generalization to other populations and dynamic tasks requires caution.

## Figures and Tables

**Table 1:** Shoulder flexion torque at different elevation angles: Actual and normalized mean with SD values. 95% confidence intervals for the mean normalized torque at each elevation angle are presented in Figure 3. All 14 participants completed valid torque trials at every elevation angle.

	Actual Values		Normalized Values	
Elevation Angle (°)	Mean (Nm)	SD (Nm)	Mean (%)	SD (%)
90	77.24	13.81	99.62	1.11
100	71.81	14.87	92.20	4.67
110	68.85	13.73	88.68	5.85
120	64.92	13.07	83.57	5.28
130	59.92	12.33	77.17	6.92
140	52.37	13.56	67.16	9.50
150	48.29	10.71	62.27	7.62
160	43.19	10.84	55.33	6.88

**Table 2:** Mean ± SD normalized EMG activity for Anterior Deltoid (AD), Medial Deltoid (MD), Biceps Brachii (BB), and Pectoralis Major (PM) across arm elevation angles (90–160°). EMG values are expressed as a percentage of each muscle’s reference contraction (MVC for MD, BB, and PM, and the 90° task-specific reference for AD), with each cell showing group mean ± SD for the specified angle and muscle. n indicates valid EMG recordings per angle and muscle; trials with artifacts were excluded.

Normalized EMG Activity				
Elev. Angle (°)	AD	MD	BB	PM
90	100.0 (-)	87.5 ± 19.9	75.7 ± 28.0	68.9 ± 24.2
90 (n)	n = 12	n = 12	n = 12	n = 7
100	98.5 ± 20.0	94.4 ± 25.3	76.4 ± 34.7	61.2 ± 20.8
100 (n)	n = 13	n = 12	n = 11	n = 7
110	102.0 ± 15.1	95.8 ± 23.0	80.7 ± 30.7	55.1 ± 22.2
110 (n)	n = 12	n = 12	n = 12	n = 7
120	101.5 ± 26.2	102.2 ± 25.4	85.1 ± 52.1	53.1 ± 26.2
120 (n)	n = 13	n = 12	n = 12	n = 7
130	98.8 ± 23.9	101.9 ± 22.7	83.5 ± 36.4	41.0 ± 23.8
130 (n)	n = 13	n = 12	n = 12	n = 7
140	95.3 ± 25.9	102.6 ± 31.6	75.9 ± 37.4	30.1 ± 24.1
140 (n)	n = 13	n = 12	n = 12	n = 7
150	97.2 ± 23.9	109.4 ± 25.6	75.4 ± 48.6	34.2 ± 32.1
150 (n)	n = 12	n = 11	n = 11	n = 7
160	99.6 ± 21.5	102.9 ± 27.8	73.8 ± 47.4	19.8 ± 5.6
160 (n)	n = 12	n = 12	n = 12	n = 6

## Data Availability

The raw data supporting the conclusions of this article will be made available by the authors on request.

## References

[1] European Agency for Safety and Health at Work (2019). Workplace Safety and Health in the EU: Trends and Statistics. https://osha.europa.eu/en/facts-and-figures.

[2] Dickerson C.R., Brookham R.L., Chopp J.N. (2011). The working shoulder: Assessing demands, identifying risks, and promoting healthy occupational performance. Phys. Ther. Rev..

[3] Anderson V., Bernard B., Burt S.E., Cole L.L., Estill C., Fine L.J., Grant K., Gjessing C., Jenkins L., Hurrell J.J. (1997). Musculoskeletal Disorders and Workplace Factors: A Critical Review of Epidemiologic Evidence for Work-Related Musculoskeletal Disorders of the Neck, Upper Extremity, and Low Back.

[4] Dickerson C.R., McDonald A.C., Chopp-Hurley J.N. (2023). Between Two Rocks and in a Hard Place: Reflecting on the Biomechanical Basis of Shoulder Occupational Musculoskeletal Disorders. Hum. Factors.

[5] Alexandrov A., Morton A., Molino J., Pelusi J., Chrostek C.A., Crisco J.J., Arcand M.A. (2025). Investigating the Effect of Elevation and Sex-Based Differences on Shoulder Proprioceptive Accuracy. Orthop. J. Sports Med..

[6] Suprak D.N., Sahlberg J.D., Chalmers G.R., Cunningham W. (2016). Shoulder elevation affects joint position sense and muscle activation differently in upright and supine body orientations. Hum. Mov. Sci..

[7] Moghadam A.N., Mohammadi R., Arab A.M., Kazamnajad A. (2011). The effect of shoulder core exercises on isometric torque of glenohumeral joint movements in healthy young females. J. Res. Med. Sci..

[8] Hecker A., Aguirre J., Eichenberger U., Rosner J., Schubert M., Sutter R., Wieser K., Bouaicha S. (2021). Deltoid muscle contribution to shoulder flexion and abduction strength: An experimental approach. J. Shoulder Elb. Surg..

[9] Chow A.Y., Dickerson C.R. (2009). Shoulder strength of females while sitting and standing as a function of hand location and force direction. Appl. Ergon..

[10] Michiels I., Bodem F. (1992). The deltoid muscle: An electromyographical analysis of its activity in arm abduction in various body postures. Int. Orthop..

[11] Bravi M., Fossati C., Giombini A., Mannacio E., Borzuola R., Papalia R., Pigozzi F., Macaluso A. (2023). Do the testing posture and the grip modality influence the shoulder maximal voluntary isometric contraction?. J. Funct. Morphol. Kinesiol..

[12] Bober T., Kulig K., Burnfield J.M., Pietraszewski B. (2002). Predictive torque equations for joints of the extremities. Acta Bioeng. Biomech..

[13] Grazi L., Trigili E., Fiore M., Giovacchini F., Sabatini A.M., Vitiello N., Crea S. (2024). Passive shoulder occupational exoskeleton reduces shoulder muscle coactivation in repetitive arm movements. Sci. Rep..

[14] Ackland D.C., Pak P., Richardson M., Pandy M.G. (2008). Moment arms of the muscles crossing the anatomical shoulder. Am. J. Anat..

[15] Ackland D.C., Pandy M.G. (2009). Lines of action and stabilizing potential of the shoulder musculature. Am. J. Anat..

[16] Inoue S., Sato M., Takahashi Y., Tamura M., Ozaki H., Furuya K., Nishinaka N. (2025). Investigation of the association between shoulder flexion range of motion and deltoid and trapezius muscle activity after reverse total shoulder arthroplasty. J. Phys. Ther. Sci..

[17] Landin D., Thompson M., Jackson M.R. (2017). Actions of the Biceps Brachii at the Shoulder: A Review. J. Clin. Med. Res..

[18] Krainak D.M., Ellis M.D., Bury K., Churchill S., Pavlovics E., Pearson L., Shah M., Dewald J.P. (2011). The effects of body orientation on maximum voluntary arm torques. Muscle Nerve.

[19] Beardsley C.L., Howard A.B., Wisotsky S.M., Shafritz A.B., Beynnon B.D. (2010). Analyzing glenohumeral torque–rotation response in vivo. Clin. Biomech..

[20] Meskers C., van der Helm F., Rozendaal L., Rozing P. (1997). In vivo estimation of the glenohumeral joint rotation center from scapular bony landmarks by linear regression. J. Biomech..

[21] Koepke N., Zwahlen M., Wells J.C., Bender N., Henneberg M., Rühli F.J., Staub K. (2017). Comparison of 3D laser-based photonic scans and manual anthropometric measurements of body size and composition. PeerJ.

[22] Winter D.A. (2009). Biomechanics and Motor Control of Human Movement.

[23] Stegeman D.F., Hermens H.J. (2007). Standards for Surface Electromyography: The European Project Surface EMG for Non-Invasive Assessment of Muscles (SENIAM).

[24] Katsavelis D., Threlkeld A.J. (2014). Quantifying thigh muscle co-activation during isometric knee extension contractions: Within- and between-session reliability. J. Electromyogr. Kinesiol..

[25] Gorgey A.S., Dudley G.A. (2008). The role of pulse duration and stimulation duration in maximizing the normalized torque during neuromuscular electrical stimulation. J. Orthop. Sports Phys. Ther..

[26] Alshatti T.A., Summers S., Hankin F.M., Kulig K. (2025). Accuracy of two methods in estimating target muscle force during shoulder submaximal isometric contractions. Int. J. Sports Phys. Ther..

[27] Papotto B.M., Rice T., Malone T., Butterfield T., Uhl T.L. (2016). Reliability of isometric and eccentric isokinetic shoulder external rotation. J. Sport Rehabil..

[28] Calver R., Cudlip A., Dickerson C.R., Mondal P., Butcher S., Kim S.Y. (2023). A comparison of isometric and isokinetic normalization methods for electromyographic data from sub-regions of supraspinatus and infraspinatus during dynamic tasks. Int. Biomech..

[29] Burden A. (2010). How should we normalize electromyograms obtained from healthy participants? What we have learned from over 25 years of research. J. Electromyogr. Kinesiol..

[30] Ito Y., Matsumoto H., Ishida T., Suenaga N., Oizumi N. (2023). Electromyographic activities of glenohumeral joint muscles during shoulder forward flexion with isometric horizontal abduction loading. J. Shoulder Elb. Surg..

[31] Ashworth B., Hank M., Khaiyat O., Coyles G., Miratsky P., Verbruggen F.F., Zahalka F., Maly T. (2025). Muscle activity relationships during isometric shoulder internal and external rotation using the ForceFrame dynamometer and athletic shoulder tests in baseball athletes. Front. Physiol..

[32] Hug F., Goupille C., Baum D., Raiteri B.J., Hodges P.W., Tucker K. (2015). Nature of the coupling between neural drive and force-generating capacity in the human quadriceps muscle. Proc. R. Soc. B Biol. Sci..

[33] Wang Z., Persad L.S., Binder-Markey B.I., Hoffman E.M., Litchy W.J., Shin A.Y., Kaufman K.R., Lieber R.L. (2025). In vivo human gracilis muscle active force-length relationship is explained by the sliding filament theory. J. Physiol..

[34] De Wilde L., Audenaert E., Barbaix E., Audenaert A., Soudan K. (2002). Consequences of deltoid muscle elongation on deltoid muscle performance: A computerised study. Clin. Biomech..

[35] Elder A., Powers C.M. (2025). Scapular Stabilization for Shoulder Pain: Putting the Cart Before the Horse?. Int. J. Sports Phys. Ther..

[36] Lieber R.L. (2002). Skeletal Muscle Structure, Function, and Plasticity.

[37] Bosch T., van Eck J., Knitel K., de Looze M. (2016). The effects of a passive exoskeleton on muscle activity, discomfort and endurance time in forward bending work. Appl. Ergon..

[38] di Luzio F.S., Tamantini C., Di Maro R., Carnazzo C., Spada S., Draicchio F., Zollo L. (2025). Biomechanical and physiological effects of passive upper limb exoskeletons in simulated manufacturing tasks. Wearable Technol..

[39] Gurney A.B., Mermier C., LaPlante M., Majumdar A., O’Neill K., Shewman T., Gurney J.G. (2016). Shoulder Electromyography Measurements During Activities of Daily Living and Routine Rehabilitation Exercises. J. Orthop. Sports Phys. Ther..

[40] Yang J., Lee J., Lee B., Kim S., Shin D., Lee Y., Lee J., Han D., Choi S. (2014). The effects of elbow joint angle changes on elbow flexor and extensor muscle strength and activation. J. Phys. Ther. Sci..

